# Evidence for different mechanisms of ‘unhooking’ for melphalan and cisplatin-induced DNA interstrand cross-links *in vitro* and in clinical acquired resistant tumour samples

**DOI:** 10.1186/1471-2407-12-436

**Published:** 2012-09-28

**Authors:** Victoria J Spanswick, Helen L Lowe, Claire Newton, John P Bingham, Alessia Bagnobianchi, Konstantinos Kiakos, Charles Craddock, Jonathan A Ledermann, Daniel Hochhauser, John A Hartley

**Affiliations:** 1 CR-UK Drug-DNA Interactions Research Group, UCL Cancer Institute, Paul O’Gorman Building, 72 Huntley Street, London, WC1E 6BT, UK; 2 Centre for Clinical Haematology, Queen Elizabeth Hospital, Birmingham B15 2TH, UK; 3 CR-UK/UCL Cancer Clinical Trials Centre, London, UK

**Keywords:** DNA interstrand cross-linking, Acquired drug resistance, DNA repair, DNA cross-linking agent, Melphalan, Cisplatin, Multiple myeloma, Ovarian cancer, DNA damage response

## Abstract

**Background:**

DNA interstrand cross-links (ICLs) are critical lesions produced by several cancer chemotherapy agents including platinum drugs and nitrogen mustards. We have previously shown in haematological (multiple myeloma) and solid tumours (ovarian cancer) that clinical sensitivity to such agents can result from a defect in DNA ICL processing leading to their persistence. Conversely, enhanced repair can result in clinical acquired resistance following chemotherapy. The repair of ICLs is complex but it is assumed that the ‘unhooking’ step is common to all ICLs.

**Methods:**

Using a modification of the single cell gel electrophoresis (Comet) assay we measured the formation and unhooking of melphalan and cisplatin-induced ICLs in cell lines and clinical samples. DNA damage response in the form of γ-H2AX foci formation and the formation of RAD51 foci as a marker of homologous recombination were also determined. Real-time PCR of 84 genes involved in DNA damage signalling pathways was also examined pre- and post-treatment.

**Results:**

Plasma cells from multiple myeloma patients known to be clinically resistant to melphalan showed significant unhooking of melphalan-induced ICLs at 48 hours, but did not unhook cisplatin-induced ICLs. In ovarian cancer cells obtained from patients following platinum-based chemotherapy, unhooking of cisplatin-induced ICLs was observed at 48 hours, but no unhooking of melphalan-induced ICLs. *In vitro*, A549 cells were proficient at unhooking both melphalan and cisplatin-induced ICLs. γ-H2AX foci formation closely followed the formation of ICLs for both drugs, and rapidly declined following the peak of formation. RPMI8226 cells unhooked melphalan, but not cisplatin-induced ICLs. In these cells, although cross-links form with cisplatin, the γ-H2AX response is weak. In A549 cells, addition of 3nM gemcitabine resulted in complete inhibition of cisplatin-induced ICL unhooking but no effect on repair of melphalan ICLs. The RAD51 foci response was both drug and cell line specific. Real time PCR studies highlighted differences in the damage response to melphalan and cisplatin following equi-ICL forming doses.

**Conclusions:**

These data suggest that the mechanisms by which melphalan and cisplatin-induced ICLs are ‘unhooked’ *in vitro* are distinct, and the mechanisms of clinical acquired resistance involving repair of ICLs, are drug specific.

## Background

DNA cross-linking agents such as the nitrogen mustards (melphalan, chlorambucil, cyclophosphamide, ifosfamide), platinum drugs (cisplatin, carboplatin, oxaliplatin), chloroethylnitrosoureas (carmustine, lomustine), the alkylalkane sulphonate busulphan and the natural product mitomycin C are widely used drugs as both single agents (including in a high-dose setting) and as components of many combination chemotherapy regimens [1,2]. In addition, more selective cross-linking agents such as SJG-136 (SG2000) continue to be developed [3,4]. Bi-functional covalent modification (cross-linking) of DNA is essential for antitumor activity with these drugs [5,6]. In particular, the DNA interstrand cross-link (ICL) which covalently links together bases on opposite strands of the DNA helix, and which normally only account for a small proportion (1-10%) of total DNA adducts, present a serious threat to cellular survival because they inhibit fundamental processes such as DNA replication and transcription [1,7]. There is clear evidence that the formation and subsequent persistence of ICLs correlates with *in vitro* cytotoxicity [8,9].

In a previous study, using a modification of the single cell gel electrophoresis (Comet) assay, we measured formation and repair of DNA ICLs in plasma cells from melphalan-naïve and melphalan-treated patients (i.e. those who had relapsed after a melphalan-conditioned autologous stem cell transplant or oral melphalan therapy) [9]. Similar levels of dose-dependent DNA ICLs were observed in cells from both melphalan-naïve and treated patients. However, marked differences in ICL repair were observed: cells from naïve patients had no repair, whereas those from treated patients exhibited between 42-100% repair at 40 hours. *In vitro* sensitivity to melphalan in plasma cells was found to correlate with ICL repair. These findings suggest that a defect in ICL repair may contribute to the initial sensitivity to melphalan and that ICL repair may be an important mechanism by which melphalan acquired resistance emerges in the clinic [10,11].

In a second study we examined ICL formation and repair in tumour cells isolated from fifty ovarian cancer patients [12]. No significant difference in the peak level of ICL formation in tumour cells was observed between patients who were either newly diagnosed, or previously treated with, platinum-based chemotherapy (or between tumour and mesothelial cells from the same patient). In contrast, the repair of ICLs was much greater in the group of treated patients. In eight patients it was possible to obtain tumour samples prior to any chemotherapy, and also at relapse or at interval de-bulking surgery following platinum chemotherapy. In these patients the mean % repair prior to therapy was 2.85 rising to 71.23 following treatment. These data again suggest that inefficient repair of ICLs contributes to the initial clinical sensitivity, and that increased ICL repair contributes to clinical acquired resistance.

Repair of ICLs is complex and requires the concerted action of multiple pathways [7,13,14]. Although the exact molecular mechanisms have yet to be fully elucidated, it is clear that incision around the lesion to allow ‘unhooking’ of the ICL from one of the two DNA strands represents a pivotal step in the repair process as it relieves the torsional stress an ICL imposes on the DNA helix and permits processing of the repair intermediates by downstream pathways. This is the step in ICL repair that can be measured using the modification of the comet assay since it detects the ability of the DNA strands to separate under alkaline conditions. A number of nucleases have been suggested to play such a role in this unhooking step, including the XPF-ERCC1 complex [5,15,16] and the Fanconi anaemia pathway orchestrates incisions at sites of crosslinked DNA (recently reviewed in [17]. Regardless of the exact mechanism of unhooking, it is widely assumed that this unhooking step will be common to all DNA ICLs. In this study, however, we present evidence *in vitro* and in clinical samples with acquired resistance that the mechanisms of unhooking for melphalan and cisplatin-induced ICLs are distinct.

## Methods

### Cell lines and peripheral blood lymphocytes

A549 and RPMI8226 cell lines were purchased from the European Collection of Cell Cultures (ECACC). The human ovarian cancer cell line A2780 was established from tumour tissue from an untreated patient [18]. Growing A2780 cells in cisplatin and selecting for cisplatin resistance generated the stably resistant A2780cisR cell line. Both cell lines were obtained from Dr Swee Sharp, Institute of Cancer Research, Sutton, UK. RPMI8226 cells was maintained in RPMI1640 media containing 2 mM L-glutamine and 10% foetal calf serum (FCS). A549 was maintained in Dulbecco’s Modified Eagles Medium (DMEM) containing 2 mM L-glutamine and 10% FCS. All cell lines were maintained in a humidified atmosphere at 37°C with 5% carbon dioxide (CO_2_) and maintained in exponential growth. The cells were kept at low passage, returning to original frozen stocks every 3 to 6 months, and tested regularly for *Mycoplasma*.

Peripheral blood lymphocytes (PBLs) were isolated using the Vacutainer® CPT™ system (Becton Dickinson, Oxford, UK). Samples were centrifuged at 1500 g for 20 minutes at room temperature. The fluffy mononuclear layer at the interface of the two layers was removed using a Pasteur pipette and transferred to a 15 ml tube. 10 ml cold RPMI 1640 tissue culture media was then added and the tube gently inverted and centrifuged immediately at 200 g for 5 minutes at 4°C. The supernatant was then discarded and the cell pellet re-suspended in RPMI 1640 containing 10% foetal calf serum and 2 mM L-glutamine.

### Patient samples

Plasma cells were isolated from bone marrow taken from Multiple Myeloma patients using standard Ficoll-Hypaque [10]. Patients 1 and 2 had relapsed following vincristine, adriamycin and dexamethasome (VAD) chemotherapy, received a melphalan conditioned (200 mg/m^2^) autologous stem cell transplant and were known to be clinically melphalan resistant. All samples studied contained in excess of 80% plasma cells.

Ovarian cancer tumour cells taken from ovarian cancer patients either pre- or post-platinum based chemotherapy, were isolated from ascitic fluid as described in detail elsewhere [12]. Ethics approval was gained from the Joint UCL/UCLH Committee on the Ethics of Human Research. In order to separate tumour cells from non-tumour mesothelial cells, ascitic fluid was centrifuged at 200 g for 5 minutes. Cell pellets were re-suspended in DMEM containing 10% FCS and 2 mM L-glutamine and seeded into large tissue culture flasks and incubated in a humidified atmosphere at 37°C with 5% CO_2_. After 1 hour, the entire volume of tissue culture medium in each flask containing unattached cells was transferred into a fresh tissue culture flask and DMEM containing 10% FCS and 2 mM L-glutamine was replaced in the original flasks. Normal mesothelial cells attached to the plastic surface within the first hour, where as tumour cells required a longer period of time to detach in response to trypsin. Further purification of the tumour samples were achieved using trypsinisation until the contaminant mesothelial cells were seen to detach, while the tumour cells remained *in situ*.

### Drug treatment

Cell lines, PBLs and patient samples were incubated with either melphalan (Sigma Chemical Co., Poole, U.K.) or cisplatin (David Bull Laboratories, Australia) for 1 hour at 37°C and 5% CO_2_ in a humidified atmosphere. For RPMI8226 cell line, PBLs and myeloma plasma cells, the drug was removed by centrifugation at 200 g for 5 minutes, the supernatant removed and cells re-suspended in drug-free full media. Cells were then incubated at 37°C and 5% CO_2_ in a humidified atmosphere. For A549, A2780, A2780cisR cell lines and patient ovarian tumour cells isolated from ascitic fluid, drug treatments were carried out in 6-well plates and the media replaced with drug free medium following treatment. In order to assess DNA interstrand cross-linking and repair, samples were taken at various time points following the 1 hour drug incubation. For combination experiments, cells were treated with 3nM gemcitabine (Eli Lilly & Company, Basingstoke, U.K.) in combination with melphalan or cisplatin. Both drugs were removed as described above and cells incubated with 3nM gemcitabine for the remainder of the incubation period.

### Determination of DNA interstrand cross-link formation and its repair using the single cell gel electrophoresis (comet) assay

The details of the single cell gel electrophoresis (comet) assay used to measure DNA interstrand cross-linking and repair are described in detail elsewhere [19]. All procedures were carried out on ice and in subdued lighting. All chemicals were obtained from Sigma Chemical Co. (Poole, U.K.) unless otherwise stated. Immediately before analysis cells were diluted to give a final concentration of 2.5 x10^4^ cells/mL and irradiated (15 Gy) in order to deliver a fixed number of random DNA strand breaks. After embedding cells in 1% agarose on a pre-coated microscope slide, the cells were lysed for 1 hour in lysis buffer (100 mM disodium EDTA, 2.5 M NaCl, 10 mM Tris–HCl pH 10.5) containing 1% Triton X-100 added immediately before analysis, and then washed every 15 minutes in distilled water for 1 hour. Slides were then incubated in alkali buffer (50 mM NaOH, 1 mM disodium EDTA, pH12.5) for 45 minutes followed by electrophoresis in the same buffer for 25 minutes at 18 V (0.6 V/cm), 250 mA. The slides were finally rinsed in neutralising buffer (0.5 M Tris–HCl, pH 7.5) then saline.

After drying the slides were stained with propidium iodide (2.5 μg/mL) for 30 minutes then rinsed in distilled water. Images were visualised using a NIKON inverted microscope with high-pressure mercury light source, 510-560 nm excitation filter and 590 nm barrier filter at x20 magnification. Images were captured using an on-line CCD camera and analysed using Komet Analysis software 4.02 (Andor Technology, U.K.). For each duplicate slide 25 cells were analysed. The tail moment for each image was calculated as the product of the percentage DNA in the comet tail and the distance between the means of the head and tail distributions [20]. DNA interstrand cross-linking was expressed as percentage decrease in tail moment compared to irradiated controls calculated by the formula:

(1) $$\%\;\text{decrease in tail moment}=\left[1-\left(\frac{\left(TMdi-TMcu\right)}{\left(TMci-TMcu\right)}\right)\right]x100$$

Where *TMdi* = tail moment of drug-treated irradiated sample; *TMcu* = tail moment of untreated, unirradiated control; *TMci* = tail moment of untreated, irradiated control.

In cells treated with DNA cross-linking agents and gemcitabine in combination, cross-linking was expressed as percentage decrease in tail moment compared to irradiated controls calculated by the formula below. This formula was used to compensate for the additional single strand breaks induced by gemcitabine in addition to those produced by the irradiation step.

(2) $$\%\;\text{decrease in tail moment}=\left[1-\left(\frac{\left(TMdi-TMcu\right)}{\left(TMci-TMcu\right)+\left(TMdu-TMcu\right)}\right)\right]x100$$

Where *TMdi* = tail moment of drug-treated irradiated sample; *TMcu* = tail moment of untreated, unirradiated control; *TMci* = tail moment of untreated, irradiated control.

In both multiple myeloma and ovarian patient samples, percentage repair was calculated at 48 hours following the peak of DNA interstrand cross-linking (9 hours for cisplatin [12] and 16 hours for melphalan [10]).

### Measurement of γ-H2AX and RAD51 foci by immunofluorescence

For the A549 cell line, 8 x 10^4^ cells per well were seeded in a 2 well LAB-TEK® II chamber slides™ (Nalgene Nunc International, Hereford, UK) and incubated overnight at 37°C. Cells were treated with either 2 μM melphalan or 5 μM cisplatin for 1 hour after which the drug was removed and cells incubated at 37°C in drug-free medium.

For the RPMI8226 cell line, cells were treated with either 2 μM melphalan or 5 μM cisplatin for 1 hour after which the drug was removed by centrifugation at 200 g, cells re-suspended and incubated at 37°C in drug-free medium. At the required time point, 10 x10^4^ cells were adhered to Vision BioSystems™ Plus slides by cytospinning at 650 rpm for 5 minutes at room temperature. Slides were then dried at room temperature.

For both cell lines, cells were fixed with ice cold methanol: acetone (50:50) for 15 min at 4°C. Cells were washed 3 times with cold PBS then permeabilized with 0.5% Triton X-100 in PBS for 15 min at room temperature. Cells were then blocked overnight at 4°C with blocking buffer (0.1% Triton X-100, 0.2% skimmed dry milk in PBS). Blocked cells were incubated overnight at 4°C with either anti-phospho-histone H2A.X (Ser139) monoclonal antibody (Millipore, U.K) at a 1:1000 dilution or anti-RAD51 (H-92) polyclonal antibody (Santa Cruz Biotechnology Inc) at a 1:100 dilution in blocking buffer. After washing 3 times with wash buffer (0.1% Triton X-100 in PBS), cells were then incubated for 4 hours at room temperature with Alexa Fluor® 488 goat anti-mouse secondary antibody (InVitrogen, UK) for γH2AX staining or Alexa Fluor® 488 goat anti-rabbit secondary antibody (InVitrogen, UK) for RAD51 staining, at a dilution of 1:1000 and 1:200 respectively in blocking buffer. Cells were then washed with PBS. For γ-H2AX, cells were counterstained with 2 μg/mL propidium iodide for 2 min. Slides were then rinsed in distilled water for 30 minutes, mounted with Vectashield® (Vector Laboratories, Peterborough, UK) and the edges sealed with clear nail polish. For RAD51, slides were mounted with Vectashield® with DAPI and the edges sealed with clear nail varnish. Images were visualised using Perkin Elmer Ultraview ERS Suite v 3.0.0 and confocal microscopy consisting of Zeiss Axiovert 200 inverted fluorescence microscope (x40 oil objective) equipped with 14 bit ECCD camera and argon and krypton gas excitation lasers at 488 nm and 568 nm. Foci were counted in 50 cells per time point and results are expressed as mean number of foci per cell from three independent experiments.

### Real-time PCR of genes involved in DNA damage signalling pathways

Exponentially growing cells were treated for 1 hour with either 150 μM cisplatin or 50 μM melphalan after which the drug was removed and replaced with drug free media. Cells were then incubated for 9 hours (cisplatin) and 16 hours (melphalan) post-treatment to allow maximum formation of interstrand cross-links. Cells were then trypsinised, washed with PBS and pelleted and stored at -80°C prior to analysis.

Total RNA was extracted from the cell pellets using a RNEasy kit (Qiagen) according to the manufacturers protocol and concentration measured. Template cDNA was generated from 1 μg of RNA using the RT^2^ First Strand Kit (SABiosciences-Qiagen). This template cDNA was then amplified in 25 μl volumes using the DNA Damage Signalling Pathway PCR Array and RT^2^ qPCR Mastermix (SABiosciences-Qiagen). Amplification was carried out in an Applied Biosystems 7500 RT-PCR machine. The RT-PCR condition was an initial incubation at 95°C for 10 minutes followed by 45 cycles at 95°C (15 seconds) and 60°C (1 minute).

Cycle Threshold (CT) values were automatically calculated using Applied Biosystems SDS software and changes in gene expression were then analysed using the online web application at http://www.sabiosciences.com/pcr/arrayanalysis.php.

The Functional Gene Groupings and Gene Table are shown in Supplementary Material.

## Results and discussion

### Different mechanisms of ‘unhooking’ of DNA ICLs in clinical acquired resistant tumour samples

**Figure 1:**
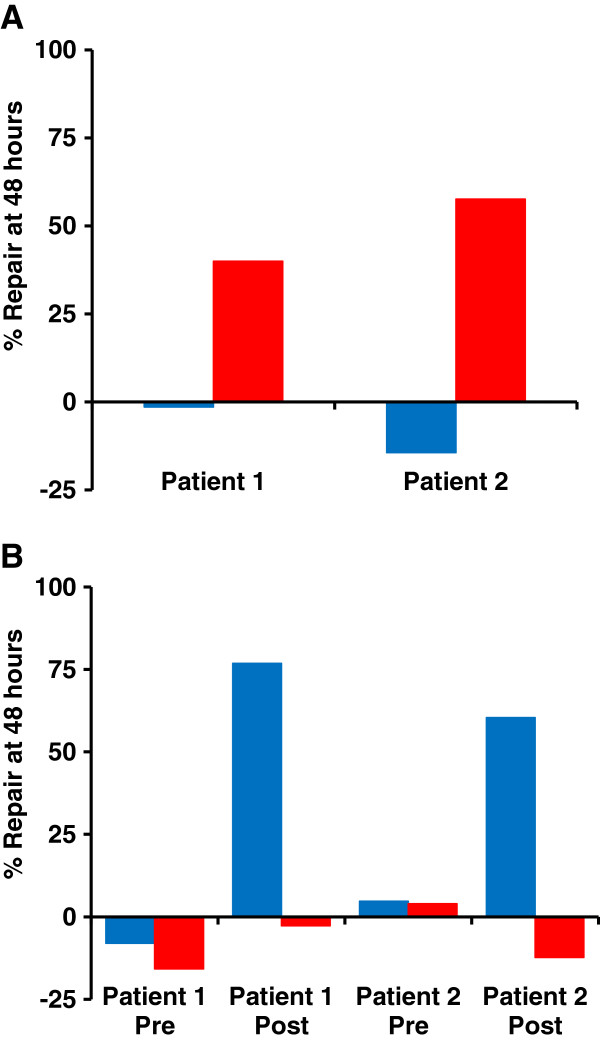
**Unhooking of DNA ICLsproduced by melphalan orcisplatin in clinical samplesmeasured using the singlecell gel electrophoresis (comet)assay.A**: Plasma cells from two multiple myeloma patients clinically resistant to melphalan. Cells were treated *ex vivo* for 1 hour with either 150 μM cisplatin or 50 μM melphalan after which the drug was removed and replaced with drug free media. Cells were then incubated for 9 hours (cisplatin, blue) or 16 hours (melphalan, red) post-treatment to allow maximum formation of interstrand cross-links. The level of ICL at this time point was compared to a second sample incubated for a total of 48 hours to obtain the % repair. **B**: As above but in tumour samples from ovarian cancer patients. In this case the two patients provided samples on initial diagnosis and prior to any platinum-based chemotherapy (Pre) and again following relapse on platinum-based therapy (Post).

We have previously shown that plasma cells from myeloma patients prior to any chemotherapy treatment are defective in ‘unhooking’ melphalan-induced ICLs when treated *ex vivo*, whereas cells from patients following treatment who become clinically resistant to melphalan are proficient in unhooking melphalan ICLs [10]. We examined the ability of plasma cells from two representative melphalan resistant patients to unhook the ICLs produced by cisplatin, in addition to melphalan (Figure 1A). Cells were treated *ex vivo* with either melphalan (50 μM) or cisplatin (150 μM) for 1 hour, drug removed and the level of ICLs measured with time using the established modification of the single cell gel electrophoresis (comet) assay [19]. The decrease in level of ICLs at 48 hour (expressed as the % repair at 48 hours in Figure 1A) was compared to the 16 hour (melphalan) and 9 hour (cisplatin) levels, which we have previously shown to be the time of peak ICL for these agents [10,12]. In both these patient samples a significant repair (unhooking) of melphalan-induced ICLs was observed (40% in patient 1 and 58% in patient 2), as has been observed previously [10]. In contrast, no unhooking of cisplatin-induced ICLs was observed at 48 hours in either patient sample. In fact, in the cells from patient 2, the level of ICLs at 48 hours was slightly higher than at 9 hours resulting in the negative ‘repair’ value.

In a further two patient samples that showed 30% and 14% unhooking of melphalan-induced ICLs, no unhooking of cisplatin-induced ICLs was observed. Cells from a melphalan naïve patient showed no repair of ICLs produced by either drug.

The ability of cancer cells taken from two ovarian cancer patients to unhook the ICLs produced by the two drugs was then examined. In these patients it was possible to obtain tumour samples at initial diagnosis (before any chemotherapy) and then again after the patients had undergone platinum-based chemotherapy. Under the identical drug-treatment conditions used in Figure 1A, the initial cells (Pre) from neither patient were able to unhook the ICLs produced by melphalan or cisplatin up to 48 hours (Figure 1B). In contrast, the tumour cells from both patients taken after platinum-based chemotherapy (Post) showed efficient unhooking of cisplatin ICLs (77% and 60% at 48 hours) but *no* unhooking of melphalan ICLs.

In total, 12 pre-chemotherapy and 7 post platinum-based chemotherapy patient samples were tested for unhooking of melphalan ICLs. The mean % repair (unhooking) was 4% and 3% for the pre- and post-chemotherapy patients, respectively. This is in marked contrast to our previously reported data where mean% repair of cisplatin ICLs was 3% pre-chemotherapy and 71% post platinum-based chemotherapy [12]. Taken together, these data suggest that distinct mechanisms are evoked in the two tumour types in patients following chemotherapy, resulting in different mechanisms of unhooking for melphalan and cisplatin-induced ICLs.

### Differences in unhooking of melphalan and cisplatin-induced ICLs in human tumour cell lines

**Figure 2:**
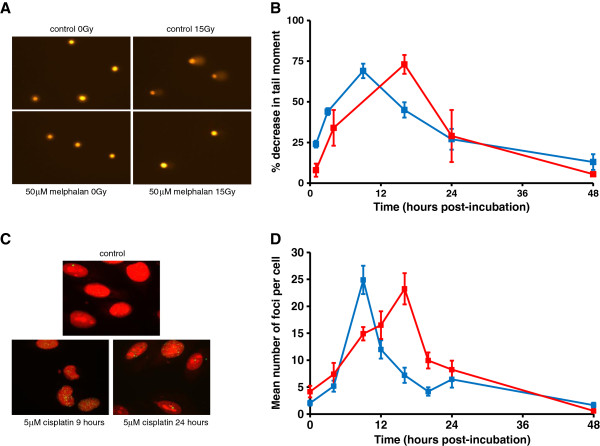
**DNA ICL and γ-H2AXfoci formation in A549cells following treatment witheither cisplatin or melphalan.A**: Representative comet images from A549 cells either untreated, or treated with 50 μM melphalan for 1 hour. Drug-treated samples shown were following a subsequent 16 hours incubation in drug free medium. **B**: Cells were treated for 1 hour with either 150 μM cisplatin (blue) or 50 μM melphalan (red) after which the drug was removed and replaced with drug free media. Samples were taken at different times of post-incubation and ICLs measured using the comet assay. Data are the mean ± s.d. from at least three independent experiments. **C**: Representative A549 cells showing γ-H2AX foci following treatment with cisplatin at 5 μM followed by post-incubation in drug free medium for the times shown. **D**: Cells were treated with either 2 μM melphalan (red) or 5 μM cisplatin (blue) for 1 hour after which the drug was removed and cells incubated at 37°C in drug-free medium. Samples were taken at different times of post-incubation and γ-H2AX foci formation determined. Data are the mean ± s.d. from at least three independent experiments.

We next looked for cell line models that could replicate the phenotype that we observed in the clinical samples. The time course of ICL formation and repair was examined in the human non-small cell lung cancer cell line A549 following a 1 hour treatment with 50 μM melphalan or 150 μM cisplatin. These drug doses, (which are within the range of the GI_50_ values as shown in Additional file: Table S1) were chosen to be consistent with our data in clinical samples and to give equivalent peak levels of ICL by the two agents. Representative comet images are shown in Figure 2A. In A549 cells, the peak of ICL was at 9 hours for cisplatin and 16 hours for melphalan, and in these cells the ICLs produced by both agents were efficiently unhooked, resulting in 92% and 81% repair at 48 hours, respectively (Figure 2B). In addition to measuring ICLs using the comet assay, DNA damage response in the form of γ-H2AX foci formation was also followed in the same cells (Figure 2C,D). Previous studies from our laboratory have shown that γ-H2AX foci formation can be used as a pharmacodynamic indicator of ICL formation for both nitrogen mustard and platinum-based drugs [21]. γ-H2AX is likely marking sites of double strand breaks generated after unhooking or lesion processing by structure specific endonucleases. Doses of drug used to treat cells for 1 hour were lower than those used in the comet assay due to the increased sensitivity of this assay. γ-H2AX foci formation followed the timing of ICL formation for both drugs, as shown previously in a different cell line [21], and rapidly declined following the peak of formation (Figure 2D). The decline in γ-H2AX suggests the resolution of the intermediate double strand breaks by downstream pathways e.g. homologous recombination repair, translesion DNA synthesis etc. We previously showed that γ-H2AX foci resulting from nitrogen mustard and cisplatin-induced ICLs persisted longer in homologous recombination defective cells [21].

**Figure 3:**
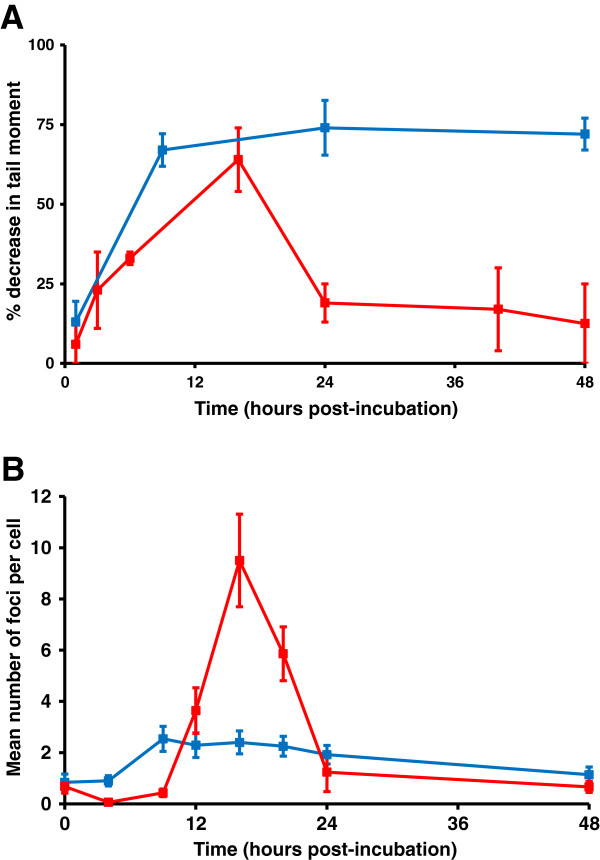
**DNA ICL and γ-H2AXfoci formation in RPMI8226cells following treatment witheither cisplatin or melphalan.A**: Cells were treated for 1 hour with either 150 μM cisplatin (blue) or 50 μM melphalan (red) after which the drug was removed and replaced with drug free media. Samples were taken at different times of post-incubation and ICLs measured using the comet assay. Data are the mean ± s.d. from at least three independent experiments. **B**: Cells were treated with either 2 μM melphalan (red) or 5 μM cisplatin (blue) for 1 hour after which the drug was removed and cells incubated at 37°C in drug-free medium. Samples were taken at different times of post-incubation and γ-H2AX foci formation determined. Data are the mean ± s.d. from at least three independent experiments.

Identical experiments were performed in the human myeloma cell line RPMI8226 (Figure 3). In this cell line the peak of melphalan-induced ICLs was again at 16 hours and significant unhooking was observed within 8 hours (Figure 3A). In contrast, cisplatin ICLs formed by 9 hours but were not unhooked over a 48 hour period. This cell line, therefore, was consistent with the phenotype seen in the melphalan-resistant plasma cells from patients (Figure 1A). This same phenotype was also observed in a second myeloma cell line U266 (data not shown). The γ-H2AX foci response in RPMI8226 cells is shown in Figure 3B. Although the response was weaker than that seen in A549 cells, γ-H2AX foci showed a similar response to melphalan, peaking with the formation of ICLs and then declining rapidly. With cisplatin, however, although cross-links form, the γ-H2AX response is extremely weak (Figure 3B). This lack of a significant DNA damage response is consistent with the lack of unhooking of the ICLs observed in this cell line, therefore preventing the subsequent generation of double strand breaks.

We then looked in human ovarian cancer cell lines for a phenotype that would mirror that observed in the clinical situation shown in Figure 1B. A2780 cells gave peak of cross-linking at 9 hours and 16 hours for cisplatin and melphalan, respectively, as seen in the other cell lines. These cells were not efficient at unhooking either type of cross-link giving 0% and 16% repair at 48 hours for cisplatin and melphalan, respectively (data not shown). This cell line therefore mirrored the clinical phenotype in chemotherapy naïve ovarian cancer (Figure 1B). A cisplatin acquired resistant line (A2780cisR) derived from A2780 was also examined. In this line the levels of ICLs produced by cisplatin and melphalan were identical to those in A2780 indicating that the mechanism of drug resistance could not be attributed to an altered transport mechanism or intracellular detoxification of the drug. This is in contract to other reports in the literature e.g. Jansen et al. 2002 [22] in which A2780cisR cells are shown to have elevated glutathione. The A2780cisR cell line used in the present study differed from the parental line, however, in that it was now capable of unhooking the cross-links produced by both agents. Since these cells efficiently unhook *both* types of cross-link, it is not representative of the phenotype observed clinically where only cisplatin-induced ICLs were repaired (Figure 1B).

p53 is one of the most important factors in determining the sensitivity of cells to DNA damage. The A549 and A2780 cell lines are both p53 wild type [23] and the RPMI8226 cell line p53 mutant [24]. Since A549 cells unhook the ICLs produced by both melphalan and cisplatin, RPMI8226 cells unhook only melphalan ICLs and A2780 unhook neither, p53 status does not explain these findings.

### Effect of gemcitabine on the unhooking of cisplatin and melphalan-induced ICLs

**Figure 4:**
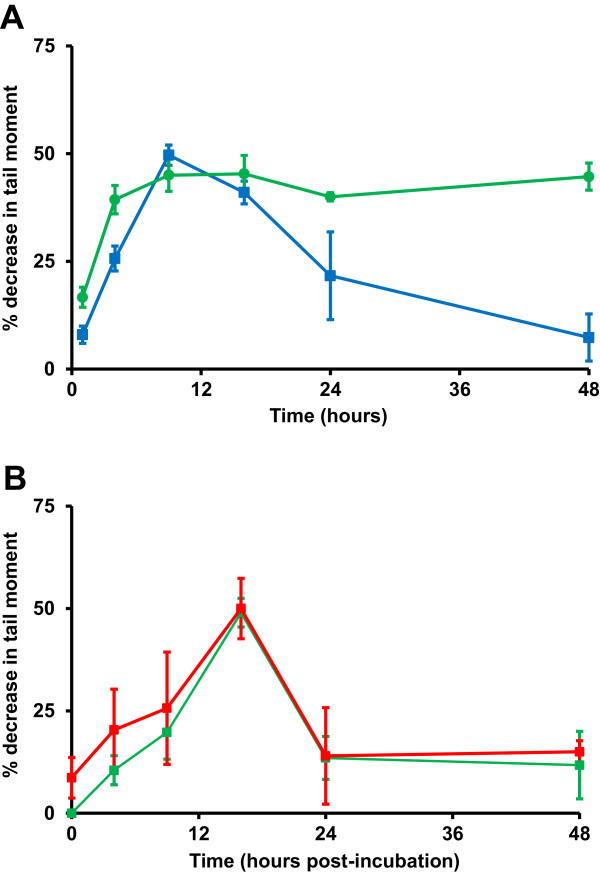
**Effect of gemcitabine onthe unhooking of cisplatinor melphalan-induced ICLs inA549 cells.A**: Cells were treated for 1 hour with 150 μM cisplatin either alone (blue) or in the presence of 3nM gemcitabine (green) after which the drugs were removed and replaced with drug free media, or media containing 3nM gemcitabine. Samples were taken at different times of post-incubation and ICLs measured using the comet assay. Data are the mean ± s.d. from at least three independent experiments. **B**: Cells were treated for 1 hour with 50 μM melphalan either alone (red) or in the presence of 3nM gemcitabine (green) after which the drugs were removed and replaced with drug free media, or media containing 3 nM gemcitabine. Samples were taken at different times of post-incubation and ICLs measured using the comet assay. Data are the mean ± s.d. from at least three independent experiments.

Gemcitabine has previously been shown to act synergistically with cisplatin *in vitro*[25] and the combination with platinum drugs is useful clinically [26-28]. We examined the effect of continuous administration of 3nM gemcitabine on the repair (unhooking) of cisplatin and melphalan-induced ICLs in A549 cells (Figure 4). In the case of cisplatin, gemcitabine completely inhibited the unhooking of ICLs with 0% repair at 48 hours compared to 85% in the absence of gemcitabine (Figure 4A). We have observed a similar inhibition of repair in lymphocytes from patients treated with the combination of carboplatin and gemcitabine [28] and fludarabine has been shown to suppress DNA ICL removal in chronic lymphocytic leukemia lymphocytes [29]. In contrast, gemcitabine at 3nM had *no* effect on the removal of melphalan-induced ICLs in A549 cells (Figure 4B). This again suggests that the mechanisms of unhooking for cisplatin and melphalan ICLs are distinct, with only the former mechanism being inhibited by gemcitabine. The mechanism by which gemcitabine inhibits the unhooking of cisplatin ICLs remains unclear. Gemcitabine is believed to inhibit nucleotide excision repair by incorporation into repair patches thereby causing chain termination. One possible mechanism is that the nucleotide excision repair of cisplatin-induced *intra*strand adducts is inhibited by incorporation of gemcitabine into repair patches resulting in sequestering of repair proteins, including those required for the initial unhooking step of DNA ICLs.

### The roles of homologous recombination and replication

**Figure 5:**
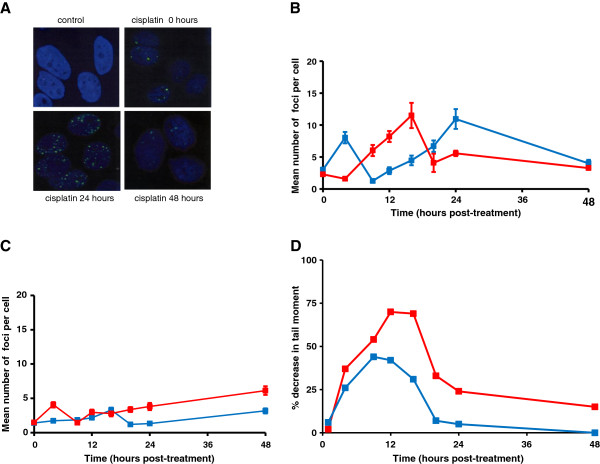
**The roles of homologousrecombination and replication inthe repair of melphalanand cisplatin-induced ICLs.A**: Representative cells showing RAD51 foci following treatment with 5 μM cisplatin followed by post-incubation in drug free medium for the times shown. **B**: A549 cells were treated with either 2 μM melphalan (red) or 5 μM cisplatin (blue) for 1 hour after which the drug was removed and cells incubated at 37°C in drug-free medium. Samples were taken at different times of post-incubation and RAD51 foci formation determined. Data are the mean ± s.d. from at least three independent experiments. **C**: RPMI8226 cells were treated with either 2 μM melphalan (red) or 5 μM cisplatin (blue) for 1 hour after which the drug was removed and cells incubated at 37°C in drug-free medium. Samples were taken at different times of post-incubation and RAD51 foci formation determined. Data are the mean ± s.d. from at least three independent experiments. **D**: Isolated human peripheral blood lymphocytes were treated for 1 hour with either 150 μM cisplatin (blue) or 50 μM melphalan (red) after which the drug was removed and replaced with drug free media. Samples were taken at different times of post-incubation and ICLs measured using the comet assay. Data are the mean from at least two independent experiments.

The formation of RAD51 foci as a marker of homologous recombination in A549 and RPMI8226 cells was examined following treatment with cross-linking agent (Figure 5). Representative RAD51 images are shown in Figure 5A. In A549 cells, a strong RAD51 foci response followed the peak of ICL for melphalan and then declined rapidly (Figure 5B), similar to the γ-H2AX response to this drug in this cell line (Figure 2D). The response following cisplatin was, however, distinct in that there was an initial peak at 4 hours with levels decreasing to baseline at 8 hours followed by a second late peak at 24 hours (Figure 5B). We have observed this biphasic response to cisplatin in other cell types including human leukaemic K562 cells and lymphocytes (data not shown). In RPMI8226 cells the RAD51 response was weak (Figure 5C), despite the formation of cross-links by both agents. Homologous recombination activity has been shown to be elevated in multiple myeloma cells leading to an increased rate of mutation and progressive accumulation of genetic variation over time [30]. Interestingly, the basal expression levels of RAD51 mRNA were 5-fold higher in RPMI8226 cells than in A549 as determined by real-time PCR (data not shown). The lack of a significant RAD51 foci response to ICLs in RPMI8226 cells is therefore not due to a lack of RAD51 protein.

It might be expected that γ-H2AX and RAD51 foci would form coincidentally. Both A549 and RPMI8226 cells can unhook melphalan-induced ICLs. In A549 cells there is a strong γ-H2AX response, peaking with the formation of ICLs and then declining rapidly. The RAD51 response follows the same time course. In contrast, in RPMI8226 cells the γ-H2AX response to melphalan ICLs is less than half that observed in A549 cells for the equivalent peak level of ICLs and in RPMI8226 cells there is no significant RAD51 response. The reasons for the different responses are unclear, however the rapid decrease in γ-H2AX foci in the RPMI8226 cells in the absence of RAD51 foci suggests that resolution of double strand breaks may not be by homologous recombination repair in these cells.

The processing of ICLs may differ in replicating and non-replicating cells [31]. We examined the ability of isolated non-replicating human lymphocytes to unhook cisplatin and melphalan-induced ICLs (Figure 5D). The peak of cross-linking for both drugs was as observed in the human tumour cell lines, and lymphocytes rapidly unhooked the cross-links produced by both drugs. Clearly, melphalan and cisplatin-induced ICLs can be unhooked in both replicating and non-replicating cells. Whether the mechanisms involved are the same is unknown and warrants further investigation.

### Expression of DNA damage response genes

**Figure 6:**
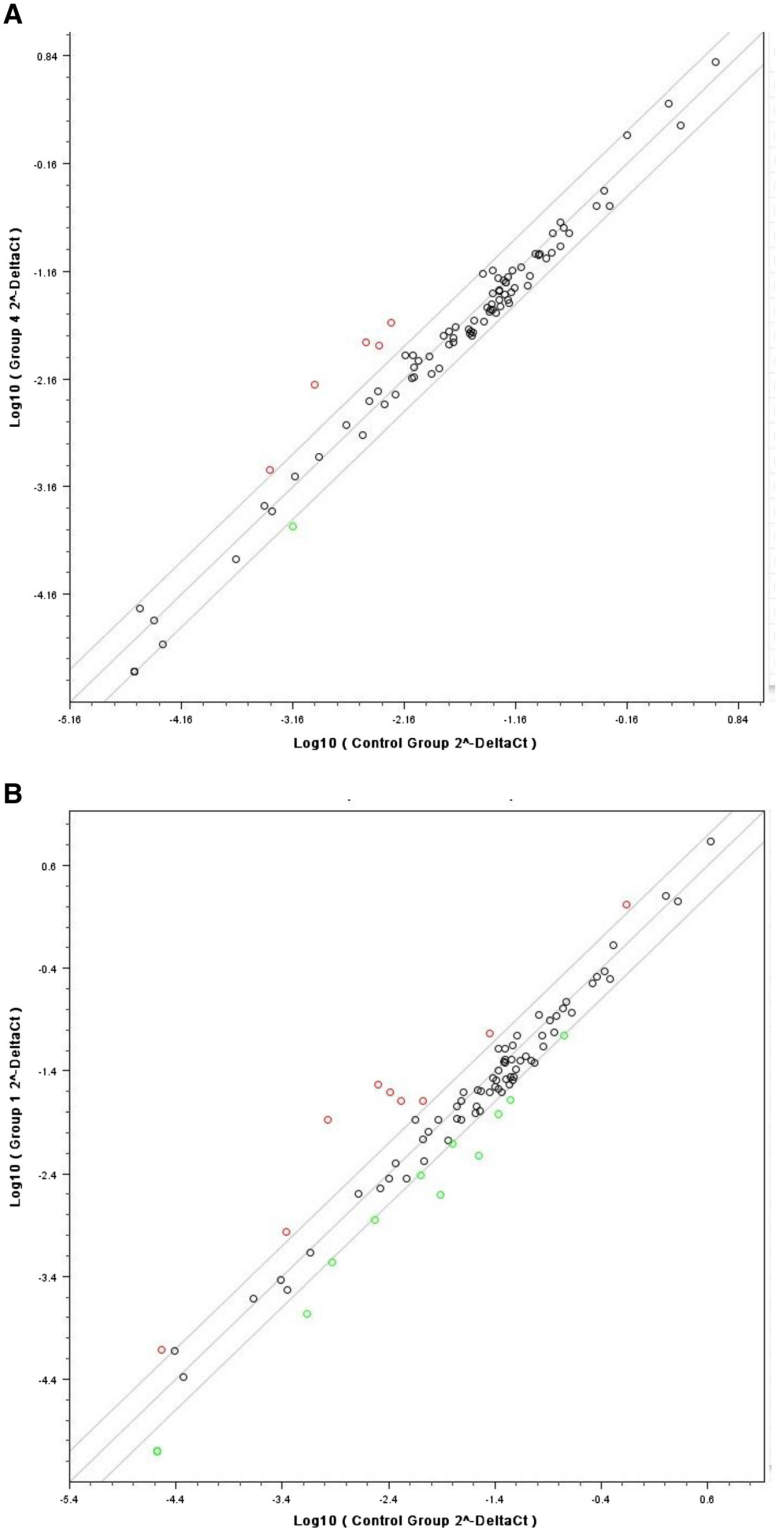
**Real time PCR analysisof the changes inexpression of 84 genesinvolved in DNA damagesignalling/repair pathways, following exposureof cells to ICLagent.A**: Prior to RNA extraction, A549 cells were treated with melphalan at 50 μM for 1 hour followed by 16 hours post-incubation to allow peak ICL formation. The mean expression from three individual drug treatment repeat samples are compared to three individual non-drug treated control samples. Genes which show increased expression by more than 2-fold compared to control cells following drug treatment are shown in red and those which show decreased expression in green. **B**: As above, but A549 cells were treated with cisplatin at 150 μM for 1 hour followed by 9 hours post-incubation to allow peak ICL formation.

In order to look for possible factors which could explain the different repair response we next examined by real time PCR the effect on expression of 84 genes involved in DNA damage signalling/repair pathways following exposure of cells to ICL agent. Comparisons were made at doses and times which gave an equivalent peak of ICL (cisplatin: 150 μM, 1 hour followed by 9 hours post-incubation; melphalan 50 μM, 1 hour followed by 16 hours). Figure 6 shows the results for A549 cells following melphalan (A) or cisplatin (B) treatment. In each case the mean expression from three individual drug treatment repeat samples are compared to three control samples. Genes which show increased expression by more than 2-fold compared to control cells following drug treatment are shown in red and those which show decreased expression in green. The genes that show more than 2-fold altered expression in A549 cells and RPMI8226 cells are detailed in Table 1. In A549 cells only five genes (BTG2, HUS1, LIG1, SESN1 and TREX1) show a greater than 2-fold increased expression following melphalan (Figure 6A, Table 1). In this cell line the same five genes exhibit increased expression after cisplatin treatment (Figure 6B, Table 1) but, in addition, four other genes (GAD45A, PCBP4, PCNA, XPC) showed increased expression. The two genes showing the greatest level of increased expression (SESN1 and BTG2) were the same for the two drugs. The number of genes that are decreased in expression by more than 2-fold (green) was much greater for cisplatin (13) compared to melphalan (1).

**Table 1:** Summary of the genes from A549 or RPMI8226 cells whose expression is changed more than two-fold following peak ICL formation by ether melphalan or cisplatin treatment compared to untreated controls

**Gene**	**Fold Regulation**			
	**A549 cells**		**RPMI8226 cells**	
	**Melphalan (50μM)**	**Cisplatin (150μM)**	**Melphalan (50μM)**	**Cisplatin (150μM)**
BTG2	**4.86**	**9.29**	*−2.06*	−1.35
HUS1	**2.28**	**2.49**	1.12	*−2.77*
LIG1	**4.43**	**3.88**	1.25	1.13
SESN1	**5.71**	**12.47**	1.50	−1.18
TREX1	**3.41**	**5.89**	*−2.75*	−1.27
DMC1	*−2.41*	*−3.97*	1.14	*−2.16*
GADD45A	1.39	**2.43**	1.64	1.88
PCBP4	1.76	**2.66**	*−4.30*	*−3.21*
PCNA	1.84	**2.40**	**2.58**	**3.63**
XPC	1.88	**2.61**	−1.60	−1.25
ATM	−1.41	*−2.09*	1.21	*−3.00*
ATRX	1.1	*−2.15*	−1.52	*−4.32*
BRCA1	−1.24	*−2.82*	−1.16	*−3.44*
CHEK2	−1.15	*−2.09*	−1.24	*−2.59*
CIDEA	−1.98	*−3.33*	*−16.32*	*−4.33*
GML	−1.98	*−3.33*	*−17.7*	*−5.77*
MAP2K6	−1.61	*−4.64*	−1.87	*−5.92*
MNAT1	−1.03	*−2.71*	−1.23	*−3.95*
MSH3	1.1	*−2.03*	1.10	*−2.75*
RAD51L1	−1.58	*−4.94*	*−3.94*	*−13.50*
SMC1A	−1.51	*−2.01*	1.10	−1.06
TP73	−1.97	*−3.33*	−1.57	*−3.38*
GTSE1	−1.64	−1.69	**3.32**	**2.63**
PRKDC	−1.47	−1.97	**2.10**	1.11
ZAK	1.06	−1.84	**3.52**	−1.51
RPL13A	−1.34	−1.18	**3.10**	**3.58**
DDIT3	1.61	1.92	*−7.33*	*−4.31*
IGHMBP2	−1.05	1.14	*−2.28*	−1.68
IP6K3	1.02	1.94	*−16.66*	*−4.79*
NBN	1.3	−1.16	*−2.18*	*−3.11*
PMS2L3	−1.56	−1.53	*−3.50*	*−3.24*
PPP1R15A	−1.43	−1.06	*−11.28*	*−3.52*
RAD17	−1.46	−1.52	*−2.21*	*−2.08*
SEMA4A	−1.97	−1.12	*−2.17*	*−3.91*
PMS1	−1.29	−1.69	*−2.01*	*−2.92*
EXO1	−1.06	1.32	1.60	**2.73**
FANCG	1.00	−1.06	1.65	**2.27**
FEN1	−1.03	1.35	1.29	**2.03**
ATR	−1.54	−1.78	−1.18	*−2.24*
CHEK1	−1.73	−1.74	−1.08	*−13.65*
MRE11A	1.19	−1.06	−1.45	*−2.78*
RAD18	−1.31	−1.61	−1.33	*−2.07*
RAD50	1.32	−1.12	−1.11	*−3.07*
XRCC2	1.24	1.23	1.05	*−3.66*

Data are the mean from three individual drug treated and three control RNA samples in each case. Genes more than two-fold upregulated are shown in **red** and more than two-fold downregulated in *green*. For comparison the fold regulation is shown for these genes in both cell lines following both treatments with downregulation shown as negative numbers.

In RPMI8226 cells the pattern of altered expression is distinct from A549 cells (Table 1). For melphalan, a different five genes had increased expression (PCNA, GTSE1, PRKDC, ZAK, RPL13A), whereas for cisplatin six genes showed increased expression (PCNA, GTSE1, RPL13A, EXO1, FANCG, FEN1). In this cell line, neither SESN1 nor BTG2 was increased by either drug. Interestingly, levels of expression of the DNA repair protein ERCC1 did not change by more than two-fold in either cell line following either cross-linking agent, despite this protein having a potential role in the unhooking step [5,15,16]. Cells defective in this protein show extreme sensitivity to both nitrogen mustard and platinum-based drugs [5,32]. Although the real time PCR data in the current study highlight differences in the damage response to melphalan and cisplatin in the two cell lines, no clear pattern emerges which could explain the different response of the cells to unhooking of cisplatin and melphalan ICLs.

## Conclusions

Overall, these data provide conclusive evidence that the mechanisms by which melphalan and cisplatin-induced ICLs are ‘unhooked’ *in vitro* are distinct. Only the latter mechanism is inhibited by gemcitabine. Importantly, the observed mechanisms of clinical acquired drug resistance in multiple myeloma to melphalan and in ovarian cancer to cisplatin, which involve repair/unhooking of ICLs, are shown to be specific to the individual drug. This clearly has important clinical implications for the treatment of drug-resistant disease.

## Abbreviation

ICL: Interstrand Cross-link.

## Competing interests

The authors declare that they have no competing interests.

## Authors’ contributions

VJS, HL, CN, and KK performed the comet assays, VJS and AB performed the γ-H2AX and RAD51 assays and JPB the real-time PCR. CC, JAL and CN provided the clinical myeloma and ovarian cancer samples. JAH conceived the study and drafted the manuscript. JAH and DH designed and directed the studies. All authors read and approved the final manuscript.

## Pre-publication history

The pre-publication history for this paper can be accessed here:

http://www.biomedcentral.com/1471-2407/12/436/prepub

## Supplementary Material

Additional file 1 **Table S1.** GI_50_ values (dose of drug that inhibits growth by 50%) for melphalan and cisplatin in the human A548, RPMI8226 cell lines. Drug exposure was for 1 hour at 37°C and cells were incubated in drug-free medium for a further 4 days prior to analysis using the sulforhodamine B assay. Values are mean±s.d. from at least three independent experiments. Click here for file
