# Supplementary material for: Evidence for different mechanisms of ‘unhooking’ for melphalan and cisplatin-induced DNA interstrand cross-links in vitro and in clinical acquired resistant tumour samples
Source: BMC Cancer. 2012 Sep 28;12:436. doi: 10.1186/1471-2407-12-436 (PMC3522549; doi:10.1186/1471-2407-12-436)
Supplement: Additional file 1 — Table S1. GI50 values (dose of drug that inhibits growth by 50%) for melphalan and cisplatin in the human A548, RPMI8226 cell lines. Drug exposure was for 1 hour at 37°C and cells were incubated in drug-free medium for a further 4 days prior to analysis using the sulforhodamine B assay. Values are mean±s.d. from at least three independent experiments. [file 1471-2407-12-436-S1.docx]

**Supplementary Table 1**

*GI_50_ values (dose of drug that inhibits growth by 50%) for melphalan and cisplatin in the human A548, RPMI8226 cell lines.* Drug exposure was for 1 hour at 37^o^C and cells were incubated in drug-free medium for a further 4 days prior to analysis using the sulforhodamine B assay. Values are mean±s.d. from at least three independent experiments.

| Cell line | GI_50_ (μM) | |
| --- | --- | --- |
|  | Melphalan | Cisplatin |
| A549 | 108 ± 21.4 | 86.8 ± 4.48 |
| RPMI8226 | 48 ± 13 | 66.7 ± 9.9 |
